# Effects of Physical Rehabilitation Integrated with Rhythmic Auditory Stimulation on Spatio-Temporal and Kinematic Parameters of Gait in Parkinson’s Disease

**DOI:** 10.3389/fneur.2016.00126

**Published:** 2016-08-11

**Authors:** Massimiliano Pau, Federica Corona, Roberta Pili, Carlo Casula, Fabrizio Sors, Tiziano Agostini, Giovanni Cossu, Marco Guicciardi, Mauro Murgia

**Affiliations:** ^1^Department of Mechanical, Chemical and Materials Engineering, University of Cagliari, Cagliari, Italy; ^2^Department of Surgical Sciences, University of Cagliari, Cagliari, Italy; ^3^AOB “G. Brotzu” General Hospital, Cagliari, Italy; ^4^Department of Life Sciences, University of Trieste, Trieste, Italy; ^5^Department of Pedagogy, Psychology, Philosophy, University of Cagliari, Cagliari, Italy

**Keywords:** rhythm, sound, RAS, Parkinson, gait, kinematics, spatio-temporal parameters, gait analysis

## Abstract

Movement rehabilitation by means of physical therapy represents an essential tool in the management of gait disturbances induced by Parkinson’s disease (PD). In this context, the use of rhythmic auditory stimulation (RAS) has been proven useful in improving several spatio-temporal parameters, but concerning its effect on gait patterns, scarce information is available from a kinematic viewpoint. In this study, we used three-dimensional gait analysis based on optoelectronic stereophotogrammetry to investigate the effects of 5 weeks of supervised rehabilitation, which included gait training integrated with RAS on 26 individuals affected by PD (age 70.4 ± 11.1, Hoehn and Yahr 1–3). Gait kinematics was assessed before and at the end of the rehabilitation period and after a 3-month follow-up, using concise measures (Gait Profile Score and Gait Variable Score, GPS and GVS, respectively), which are able to describe the deviation from a physiologic gait pattern. The results confirm the effectiveness of gait training assisted by RAS in increasing speed and stride length, in regularizing cadence and correctly reweighting swing/stance phase duration. Moreover, an overall improvement of gait quality was observed, as demonstrated by the significant reduction of the GPS value, which was created mainly through significant decreases in the GVS score associated with the hip flexion–extension movement. Future research should focus on investigating kinematic details to better understand the mechanisms underlying gait disturbances in people with PD and the effects of RAS, with the aim of finding new or improving current rehabilitative treatments.

## Introduction

Parkinson’s disease (PD) is a neurodegenerative disorder traditionally attributed to the progressive degeneration of dopaminergic neurons in the substantia nigra and, more recently, of other non-dopaminergic systems of basal ganglia and of other regions of the central nervous system (1–3). Although PD patients report both motor and non-motor symptoms, the former (tremor, rigidity, bradykinesia, postural instability, and gait disturbance) have a huge impact on daily activities and may severely reduce the patients’ quality of life. In particular, the management of gait disorders, which are frequently encountered in PD, is of crucial importance because, as the disease progresses, they result in immobility (which causes loss of independence) and risk of falling (4).

Individuals with PD typically exhibit a gait pattern characterized by short stride length, increased cadence, and reduced velocity (5), which tends to further deteriorate with the progression of the disease (6). For this reason, pharmacological therapies are not sufficient to adequately deal with gait impairments and physical therapy is essential to cope with the deterioration in motor functions. Within the physical therapy domain, in the mid-1990s the efficacy of a therapy associated with rhythmic sounds, called Rhythmic Auditory Stimulation (RAS) (7), proved to be successful.

The rationale underpinning the effectiveness of RAS interventions lies in the origin of the gait disturbance in PD. The simultaneous activation and relaxation of many muscles in a coordinated way with very high temporal precision is necessary to perform a fluent gait. In healthy humans, this process is generally performed automatically. In PD patients, the cognitive mechanisms responsible for automatically processing the temporal coordination of movements – which typically involve basal ganglia – are somehow impaired (8, 9). Indeed, empirical evidence suggests that the “internal clock” that regulates both perceptual and motor processes is affected by PD (10, 11). As a consequence, patients affected by PD generally perform poorly in cognitive tasks involving temporal processing and in the execution of automatic cycling movements, such as walking. To cope with this impairment, interventions based on RAS provide patients with an auditory temporal guidance, which facilitates the regulation of their movements while walking (12).

In one of the first studies of RAS by Thaut and colleagues (7), the researchers randomly assigned patients to one of three conditions: RAS training, internally self-paced training and no training. Even though the analysis of spatio-temporal parameters revealed improvements in both training conditions, the patients assigned to the RAS condition had significantly better results in gait velocity, stride length, and step cadence compared to the other two conditions. In the subsequent years, researchers manipulated important parameters of the original training protocol [for recent reviews, see Ref. (9, 12, 13)]. For instance, some studies investigated the immediate effects of RAS in real-time imitation tasks [e.g., Ref. (14, 15)], while other studies manipulated the duration of the training program (i.e., number of weeks, number of sessions, duration of each session), the stimuli (i.e., tempo and type of sounds), and exercises [e.g., Ref. (8, 16–23)]. Overall, the majority of these studies confirmed the efficacy of rehabilitation accompanied by RAS, in particular in terms of spatio-temporal parameters of gait (18, 19, 22, 24–26).

It is noteworthy that the effects of RAS on gait patterns of people with PD were usually assessed by analyzing changes that occurred within spatio-temporal parameters, such as velocity, cadence, and stride length (9), while other important aspects, such as kinematic parameters (i.e., joint angular displacements at ankle, knee, hip, and pelvis districts) remained mostly unexplored. The only exception is represented by the study carried out by Picelli et al. (27) who investigated the effects of cued walking at different cadences on spatio-temporal and kinematic parameters of gait, finding that auditory cues are able to improve gait through modifications of motor strategies. The fact that kinematics has been rarely investigated is quite surprising, considering that previous studies recognized the importance of investigating the kinematic profiles of gait patterns in people with PD (28). In fact, this analysis allows the identification of a number of distinctive features (i.e., flat foot contact, reductions in the range of hip extension in mid-stance, knee flexion in swing, and plantarflexion at toe push-off) (28) which are crucial when the effects of neurosurgical, pharmacological, and rehabilitative treatments must be assessed (13).

The literature reports few attempts to investigate the effectiveness of rehabilitative treatments integrated with RAS through kinematic analysis of gait in other kinds of neurological diseases, such as stroke or cerebral palsy (29–31). In particular, two studies (30, 31) assess the overall deviation from a physiologic gait pattern from a kinematic point of view using the gait deviation index (GDI), a multivariate measure of overall gait pathology based on a set of features extracted from kinematic data (32). In both cases, RAS was found to have a beneficial effect on kinematic as well as on spatio-temporal gait patterns.

Thus, on the basis of the aforementioned considerations, this study aimed to assess the effect on gait patterns of 5 weeks of rehabilitative treatment that included gait training assisted by RAS. We hypothesized that a rehabilitative protocol integrated with RAS would improve not only the spatio-temporal parameters of gait, but also the kinematics. Moreover, to investigate the possible persistence of training effects, we performed a follow-up assessment 3 months after the end of the treatment.

## Materials and Methods

### Participants

In the period from October 2014 to March 2015, 50 outpatients with PD admitted to the G. Brotzu General Hospital (Cagliari, Italy) for rehabilitation treatment were informed about the study. Assessment was carried out by a neurologist (Giovanni Cossu) experienced in PD, when patients were in “ON” state 60–90 min after intake of the usual morning l-DOPA dose. All screened patients met the PD UK Brain Bank criteria (33). The inclusion criteria for the study were as follows: ability to walk independently with no assistance; hearing capacity sufficient to perceive the auditory cues; absence of significant cognitive impairment (e.g., Mini-Mental Status Examination (MMSE) > 24; Frontal Assessment Battery (FAB) > 13); absence of psychiatric or severe systemic illnesses; mild-to-moderate disability assessed by means of the modified Hoehn and Yahr (H&Y) staging scale (1 ≤ H&Y ≤ 3). Patients were excluded if they were engaged in any training or rehabilitative program in the 3 months prior to the beginning of the study. At the time of enrollment, all participants were treated with l-DOPA and five of them were also taking dopamine agonists.

After the medical examination and an interview to establish the motivation level of potential participants, 31 individuals were included and scheduled for the treatment. The local ethics committee approved the study and all participants signed an informed consent form after a detailed explanation of the purposes of the study and of the methodology used for the experimental tests.

### Rehabilitation Protocol

Participants performed 5 weeks of supervised rehabilitative treatment (articulated in 2 × 45-min sessions/week) as outpatients at the hospital’s Physical Medicine and Rehabilitation Department. Each of them was supervised by a physical medicine specialist (Carlo Casula) and individually assisted in the training by a certified physical therapist. The typical training session included a set of exercises aimed to enhance mobility, balance, and posture as well as specific gait training (see Appendix A for details). In particular, 20 min of each session were dedicated to continuous level walking, while participants equipped with a portable MP3 player and headphones listened to the auditory cues (RAS). During this period, participants were also instructed to perform at their homes (at least three times a week) a subset of the same exercises as used at the hospital, including 30 min of gait with RAS. Patients were provided with a diary in which they self-reported both the duration and type of activities performed at home. The diary was monitored by the physical therapists twice a week.

The RAS consisted of auditory beats whose pace (beats per minute – bpm) was personalized for each participant on the basis of the first gait assessment performed before the beginning of the study. The pace, that reflects on imposed gait cadence during the training, was set on the basis of the difference between the cadence of each patient and of healthy individuals of the same age range as reported in previous studies (34, 35). In particular, for participants whose cadence at the beginning of the study was:

(a) below normality, the RAS pace was set at a value of 10% higher than one’s own cadence (e.g., if normality was 100 steps/min and the patient’s cadence was 80, the stimulus was set at 88 bpm);
(b) below, but close to normality (less than 10% difference), the RAS pace was set at normality values (e.g., if normality was 100 steps/min and the patient’s cadence was 95, the stimulus was set at 100 bpm);
(c) above normality, the RAS pace was set at values equal to one’s own cadence (e.g., if normality was 100 steps/min and the patient’s cadence was 105, the stimulus was set at 105 bpm). In any case, stimuli could not exceed 130 bpm.

At the end of the 5 weeks of supervised training, participants were instructed to perform, on a daily basis, the same exercises at home for the subsequent 12 weeks. They were invited to perform 30 min of exercises 5 days a week. This training was completely unsupervised. After this period, they were called to the laboratory for the follow-up assessment. In the follow-up, patients were interviewed by a physical medicine specialist and in general they confirmed their adherence to the training program during the unsupervised period.

### Measurement of Spatio-Temporal and Kinematic Gait Parameters

The acquisition of both spatio-temporal and kinematic gait parameters was performed at the Laboratory of Biomechanics and Industrial Ergonomics of the University of Cagliari (Italy) before the beginning of the study (T0), after its conclusion (+5 weeks, T5) and after 3 months follow-up (+17 weeks, T17) using an optoelectronic system composed of eight infrared Smart-D cameras (BTS Bioengineering, Italy) set at a frequency of 120 Hz. After anthropometric data collection, 22 spherical retroreflective passive markers (14 mm in diameter) were placed on the skin of the individual’s lower limbs and trunk at specific landmarks, following the protocol described by Davis et al. (36). Participants were then asked to walk barefoot at a self-selected comfortable speed in the most natural manner possible on a 10-m walkway for at least six times, allowing suitable rest times between the trials. The raw data were then processed with the Smart Analyzer (BTS Bioengineering, Italy) dedicated software to calculate:

- seven spatio-temporal parameters (gait speed and cadence, step length, step width, stance, swing, and double support phase duration expressed as percentage of the gait cycle);
- nine kinematic parameters, namely pelvic tilt, rotation and obliquity, hip flexion–extension, adduction–abduction and rotation, knee flexion–extension, ankle dorsi–plantarflexion, and foot progression (i.e., the angle between the axis of the foot and the walking direction);
- dynamic range of motion (ROM) for hip and knee flexion–extension and ankle dorsi–plantarflexion calculated during the whole gait cycle as the difference between the maximum and minimum value of each angle recorded during a trial.

Kinematic data were summarized using the Gait Variable Score (GVS) and the Gait Profile Score (GPS). These concise measures of gait quality were recently proposed by Baker et al. (37) as a simplification of the GDI approach previously formulated by Schwartz and Rozumalski (32): in fact, using GPS instead of GDI has some advantages, such as the reduced set of parameters considered (9 vs. 15) and the fact that GPS can be decomposed into individual joint and plane scores (GVS). This approach was found effective in characterizing gait alterations in individuals with PD (38, 39) as well as in those affected by other neurological and non-neurological diseases, thus demonstrating general validity and a broad spectrum of applications (40–42). Specifically, the GVS represents the root mean square (RMS) difference between the tested subject’s curve for a certain movement (e.g., knee flexion–extension) and a reference curve calculated as the mean value of tests performed on the unaffected subjects. The GPS combines the nine GVS values in a single score, which indicates the degree of deviation from a hypothetical “normal” gait (i.e., the larger the GPS, the less physiological the gait pattern); values for healthy individuals lie in the range of 5–6° (41). In the case of the present study, the reference data were obtained from a database of healthy individuals, of the same age range of the subjects here tested, available from the Smart Analyzer software.

### Statistical Analysis

Spatio-temporal and kinematic variables of gait were assessed before treatment with RAS (T0), at the end of treatment, i.e., 5 weeks after the baseline (T5), and 3 months after the end of treatment, i.e., 17 weeks after the baseline (T17). When different measures were available for the left and right limbs, a preliminary *t*-test was carried out to assess possible differences between them. Given that no significant differences were found for any of the investigated parameters, the mean value calculated across the two limbs was considered representative of each participant and was used for the subsequent analyses.

The independent variable was time (T0, T5, T17) and the dependent variables were the nine GVS scores plus the GPS index, the dynamic ROM of hip, knee and ankle joints in the sagittal plane, and the seven spatio-temporal parameters previously listed. To evaluate possible differences in the dependent variables across time, a set of repeated-measures analyses of variance (RM ANOVAs) was applied. When the sphericity assumption (calculated with the Mauchly’s test) was violated, data were corrected with the Greenhouse–Geisser formula. When the normality distribution assumption (calculated with the Shapiro–Wilk’s test) was violated, Friedman’s test instead of the RM ANOVA was used. When the omnibus values of RM ANOVAs and Friedman’s tests were significant, the contrasts using the paired-samples *t*-test and the Wilcoxon’s test, respectively, were calculated. The alpha level was set at 0.05 for the omnibus tests and was adjusted with the Bonferroni formula for the contrasts (0.05/3 comparisons = 0.017). The analyses were performed using the IBM SPSS Statistics v.20 software (IBM, Armonk, NY, USA).

## Results

Of the 31 patients who entered the study, 26 (20 males, 6 females) completed the training program and underwent the three gait assessments. Five participants were forced to leave the study due to musculoskeletal injuries (not related to the rehabilitative program, four cases) or chemotherapy (one case). The main anthropometric and clinical features of the 26 participants are given in Table 1.

**Table 1:** **Main features of the 26 participants**.

Parameter	Value
Age (years)	70.4 ± 9.0
PD duration (years)	7.5 ± 5.4
Hoehn and Yahr (H&Y)	1 ≤ H&Y ≤ 3
Unified Parkinson’s disease rating scale (UPDRS III)	27.3 ± 9.5
Mini-mental status examination (MMSE)	28.7 ± 1.9
Frontal assessment battery (FAB)	16.9 ± 1.4

*Values are expressed as mean ± SD*.

The effects of the physical therapy with RAS across time are separately reported for spatio-temporal and kinematic variables.

### Spatio-Temporal Parameters

**Figure 1:**
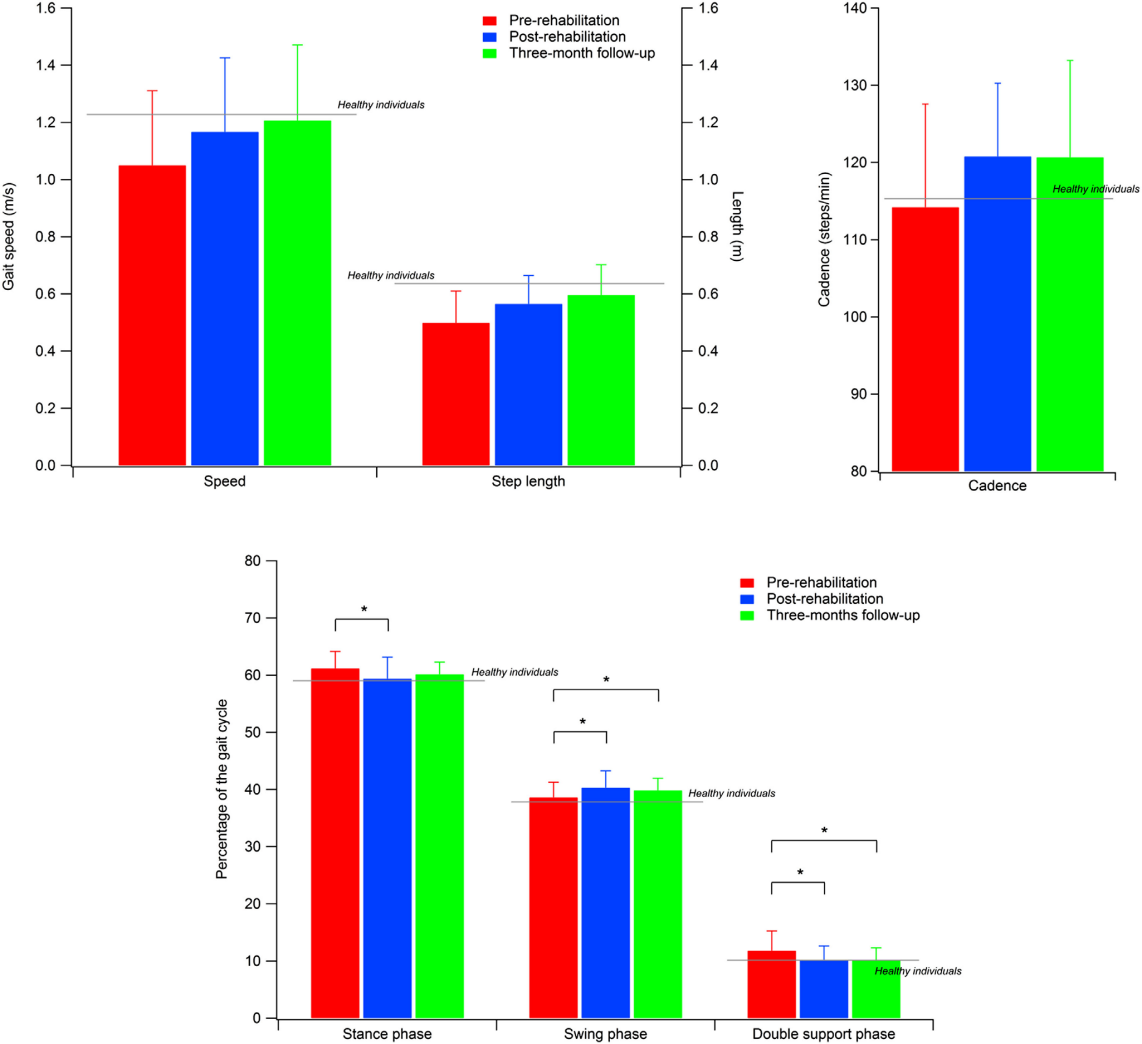
**Spatial–temporal parameters of the participants with PD**. Gray lines indicate the reference values calculated for a sample of healthy individuals. Error bars indicate SD.

The spatio-temporal parameters calculated for the three experimental conditions are shown in Table 2, while Table 3 provides the details of the cadence values for each participant at the baseline and after the rehabilitative treatment. Figure 1 shows the values of the spatio-temporal parameters of the participants compared with those calculated for an age- and gender-matched group of healthy individuals tested in the same laboratory.

**Table 2:** **Comparison between spatio-temporal parameters assessed before and after rehabilitation**.

Spatio-temporal gait parameters				
	T0	T5	T17	Time *p*-value
Step length (m)	0.50 ± 0.11	0.56 ± 0.10^a^	0.60 ± 0.10^a^^,^^b^	<0.001
Gait speed (m/s)	1.05 ± 0.26	1.16 ± 0.26^a^	1.21 ± 0.26^a^	<0.001
Cadence (steps/min)	114.56 ± 13.35	120.83 ± 9.38^a^	120.58 ± 12.29^a^	0.024
Step width (m)	0.17 ± 0.03	0.18 ± 0.03	0.20 ± 0.05^a^^,^^b^	<0.001
Stance phase (% of the gait cycle)	61.07 ± 2.75	59.41 ± 3.07^a^	60.13 ± 1.96	0.002
Swing phase (% of the gait cycle)	38.72 ± 2.56	40.30 ± 2.45^a^	39.85 ± 1.97^a^	0.004
Double support (% of the gait cycle)	11.65 ± 2.62	10.21 ± 2.07^a^	10.20 ± 1.97^a^	0.002

*Values are expressed as mean ± SD*.

*T0, baseline; T5, after 5 weeks of supervised rehabilitation; T17, 3-months’ follow-up*.

*^a^denotes statistical significance with respect to baseline*.

*^b^denotes statistical significance with respect to T5*.

**Table 3:** **Cadence values for each participant before and after rehabilitative treatment**.

Participant #	Age	Reference cadence (bpm, 34, 35)	Imposed cadence (bpm)	Cadence at T0	Cadence at T5	Cadence at T17
1	68.6	117	117	117	124	125
2	81.5	103	106	106	114	114
3	75.0	115	126	126	126	127
4	79.4	115	110	100	119	146
5	48.6	121	96	87	127	131
6	56.0	122	123	124	121	126
7	79.5	110	110	105	103	91
8	54.2	122	118	107	122	119
9	67.3	117	117	111	114	110
10	67.0	117	117	109	116	110
11	66.3	117	130	131	130	125
12	71.2	115	130	141	132	130
13	79.4	103	103	97	115	120
14	79.9	103	103	95	116	113
15	71.0	122	122	118	120	121
16	74.0	115	130	130	138	133
17	75.1	115	112	101	115	113
18	76.8	115	124	124	131	128
19	65.8	117	123	123	131	125
20	79.9	103	118	118	121	122
21	69.2	117	114	104	97	88
22	75.2	115	125	125	128	124
23	71.9	122	122	119	125	125
24	69.8	115	130	132	130	136
25	52.5	118	113	103	107	116
26	75.8	122	123	123	122	119

All parameters revealed a significant omnibus value for time: gait speed [*F*(2, 50) = 8.402; *p* < 0.001; η^2^ = 0.252]; cadence [χ^2^(2) = 7.462; *p* < 0.05; *W* = 0.143], step width [χ^2^(2) = 20.356; *p* < 0.001; *W* = 0.391], step length [*F*(2, 50) = 20.775; *p* < 0.001; η^2^ = 0.454], and percentage of swing phase [*F*(2, 50) = 6.171; *p* < 0.005; η^2^ = 0.198], double support phase [*F*(2, 50) = 7.370; *p* < 0.005; η^2^ = 0.228], and stance phase [χ^2^(2) = 13.000; *p* < 0.005; *W* = 0.250]. Thus, we were able to calculate the contrasts for all variables.

The contrasts revealed augmented gait speed [*t*(25) = 2.839; *p* < 0.005; *d* = 0.433], cadence (*Z* = 2.845; *p* < 0.005; *r* = 0.394), and percentage of swing phase [*t*(25) = 3.172; *p* < 0.005; *d* = 0.628], and reduced percentage of double support phase [*t*(25) = 3.206; *p* < 0.005; *d* = 0.604] between T0 and T5. Moreover, the data showed that these improvements were kept constant between T5 and T17, and in T17 were still significantly different from T0: gait speed [*t*(25) = 3.580; *p* < 0.001; *d* = 0.591], cadence (*Z* = 2.222; *p* < 0.05; *r* = 0.308), percentage of swing phase [*t*(25) = 2.505; *p* < 0.01; *d* = 0.483], and percentage of double support phase [*t*(25) = 2.967; *p* < 0.005; *d* = 0.618].

As for the percentage of the stance phase, we found a pattern of results similar to that of previous analyses, with significant improvements between T0 and T5 (*Z* = 2.502; *p* < 0.01; *r* = 0.347) and no difference between T5 and T17. However, in this case, the difference between T0 and T17 (*Z* = 2.007; *p* < 0.05; *r* = 0.278) was no longer significant after the Bonferroni correction. Conversely, we found that step length significantly improved between T0 and T5 [*t*(25) = 3.423; *p* < 0.001; *d* = 0.573], and had still improved in T17, both compared to T0 [*t*(25) = 6.634; *p* < 0.001; *d* = 0.871] and T5 [*t*(25) = 2.805; *p* < 0.005; *d* = 0.333]. Finally, we also found higher values in T17 compared to both T0 (*Z* = 3.672; *p* < 0.001; *r* = 0.509) and T5 (*Z* = 3.213; *p* < 0.001; *r* = 0.445) for step width. However, in this case, the difference between T0 and T5 did not reach a significant value (*p* < 0.09).

### Kinematic Parameters

Kinematic changes due to physical therapy with RAS were evaluated through the GPS, GVS, and dynamic ROM values.

#### GPS and GVS Values

**Figure 2:**
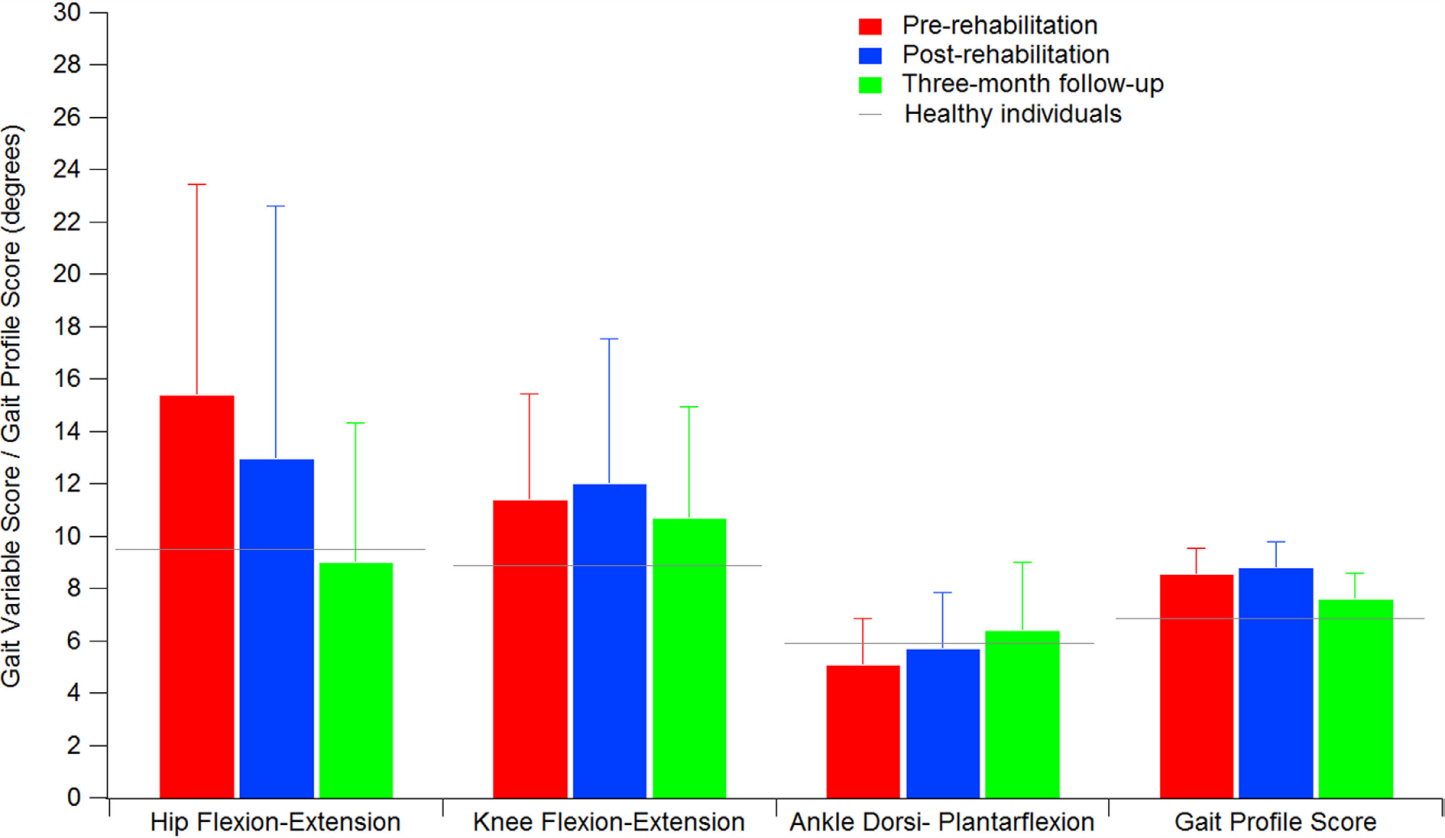
**GPS and GVS of hip, knee, and ankle in the sagittal plane of the participants with PD**. Gray lines indicate the reference values calculated for a sample of healthy individuals. Error bars indicate SD.

Higher GVS values indicate a large deviation from physiologic conditions for a specific movement of the nine previously listed; the GPS combines all the nine GVSs in a single value to summarize with a single value the overall quality of the gait pattern. The GPS and GVS scores calculated for the three experimental conditions are shown in Table 4, while Figure 2 shows the GVS calculated in the sagittal plane for hip, knee, and ankle joints and the GPS values compared with those calculated for healthy individuals of the same age range.

**Table 4:** **Comparison between kinematic parameters of gait assessed before and after rehabilitation**.

Kinematic gait parameters					
		T0	T5	T17	Time *p*-value
GVS (°)	GPS (°)	8.48 ± 2.28	8.77 ± 2.67	7.59 ± 1.72^a^^,^^b^	0.013
	Pelvic tilt	6.66 ± 4.47	6.60 ± 5.41	5.06 ± 4.01	0.112
	Pelvic rotation	3.49 ± 1.29	3.91 ± 1.17	3.73 ± 1.14	0.405
	Pelvic obliquity	2.98 ± 1.31	3.10 ± 1.21	2.84 ± 1.03	0.621
	Hip flexion–extension	14.59 ± 7.74	12.36 ± 9.06	8.56 ± 4.84^a^^,^^b^	0.006
	Hip abduction–adduction	3.79 ± 1.12	4.16 ± 1.16	3.80 ± 1.21	0.425
	Hip rotation	9.56 ± 4.34	10.71 ± 4.22	9.80 ± 3.38	0.607
	Knee flexion–extension	11.25 ± 2.76	11.77 ± 4.85	10.57 ± 3.65	0.354
	Ankle dorsi–plantarflexion	5.10 ± 1.10	5.63 ± 1.73	6.40 ± 2.13^a^	0.013
	Foot progression	7.75 ± 4.98	6.46 ± 2.91	7.63 ± 3.39	0.347

*Values are expressed as mean ± SD*.

*T0, baseline; T5, after 5 weeks of supervised rehabilitation; T17, 3-months’ follow-up*.

*^a^denotes statistical significance with respect to baseline*.

*^b^denotes statistical significance with respect to T5*.

A significant omnibus value was found for the GPS [χ^2^(2) = 8.615; *p* < 0.05; *W* = 0.166] and GVS of hip flexion–extension [χ^2^(2) = 10.272; *p* < 0.01; *W* = 0.198] and ankle dorsi–plantarflexion [*F*(2, 50) = 4.759; *p* < 0.05; η^2^ = 0.160]. The contrasts revealed lower GPS scores in T17 compared to both T0 (*Z* = 2.109; *p* < 0.05; *r* = 0.292) and T5 (*Z* = 2.502; *p* < 0.01; *r* = 0.347). Similarly, the GVS of hip flexion–extension was lower in T17 compared to both T0 (*Z* = 3.565; *p* < 0.001; *r* = 0.494) and T5 (*Z* = 2.299; *p* < 0.05; *r* = 0.330). In this case, the difference between T0 and T5 appeared to be significant (*Z* = 1.740; *p* < 0.05; *r* = 0.241), but this value was no longer significant after the Bonferroni correction. Finally, it was found that the GVS of ankle dorsi–plantarflexion in T17 was higher than in T0 [*t*(25) = 2.726; *p* < 0.05; *d* = 0.746].

#### Dynamic ROM

Differently from GVSs, higher ROM values indicate a better functionality of a certain articular joint. The ROM calculated for the three experimental conditions are shown in Table 5. The omnibus analyses revealed significant values for ROM of knee flexion–extension [χ^2^(2) = 13.000; *p* < 0.005; *W* = 0.250] and ROM of hip flexion–extension [*F*(1.2, 30.2) = 20.058; *p* < 0.001; η^2^ = 0.445]. The values for ROM of knee flexion–extension significantly improved from T0 to T5 (*Z* = 3.048; *p* < 0.001; *r* = 0.423), remained stable between T5 and T17, and were still significant at T17 compared to T0 (*Z* = 3.213; *p* < 0.001; *r* = 0.446). The hip flexion–extension ROM significantly improved from T0 to T5 [*t*(25) = 3.943; *p* < 0.001; *d* = 0.498]. In T17, the values were significantly higher compared to both T0 [*t*(25) = 5.209; *p* < 0.001; *d* = 0.620] and T5 [*t*(25) = 2.622; *p* < 0.01; *d* = 0.132].

**Table 5:** **Comparison between dynamic ROM assessed before and after rehabilitation**.

Dynamic range of motion				
	T0	T5	T17	Time *p*-value
Hip flexion–extension (°)	37.84 ± 7.38	41.35 ± 6.48^a^	42.20 ± 6.21^a^^,^^b^	<0.001
Knee flexion–extension (°)	53.59 ± 6.08	56.23 ± 5.02^a^	56.84 ± 3.98^a^	0.002
Ankle dorsi–plantarflexion (°)	24.27 ± 5.07	24.31 ± 4.21	24.60 ± 3.90	0.540

*Values are expressed as mean ± SD*.

*T0, baseline; T5, after 5 weeks of supervised rehabilitation; T17: 3-months’ follow-up*.

*^a^denotes statistical significance with respect to baseline*.

*^b^denotes statistical significance with respect to T5*.

## Discussion

The main goal of the present study was to assess the effectiveness of 5 weeks of rehabilitative treatment that included gait training assisted by RAS. The major novelty of the research is represented by the use of state-of-the-art technologies for quantitative human movement analysis to verify possible changes introduced by the treatment in the gait patterns of tested participants, especially in terms of kinematics. We also aimed to verify, after a 3-month follow-up, whether the positive effects of the training were maintained or not.

Our results confirm previous reports as regards the positive effects of RAS on spatio-temporal parameters of gait, whose results all (except step width) significantly improved at the end of the supervised treatment. In particular, in four cases out of seven (i.e., step length, gait speed, cadence, and double support phase duration), such changes were maintained at the 3-month follow-up. It is also noteworthy that the increase observed for gait speed (0.14 m/s) can be considered a large clinically meaningful effect (43). Moreover, the results obtained here show that the training resulted in a recovery of functionality characterized by post-rehabilitation/follow-up values similar to those calculated in previous studies for healthy individuals of the same age range (34, 35, 44–48) as shown for the cases of speed, step length, and cadence in Figure 1.

By contrast, it was quite surprising to observe a significant increase in the width of the base of support as a sort of “side effect” of the treatment; in fact, higher values of this parameter are usually associated with reduced stability and fear of falling (49, 50). We hypothesized that such apparently negative effects are actually due to increased speed, meaning that the individuals appeared to adapt their gait strategy to the new speed they were able to achieve by enlarging the base of support, as they felt more confident. This phenomenon was previously observed by Helbostad and Moe-Nilssen (51), who reported the existence of a “u-shaped” relationship between gait speed and step width in elderly subjects. However, this issue should be further investigated with specific tests on gait at different speeds in individuals with PD, to verify whether the same kind of trend remains in presence of the pathology.

As regards gait kinematics, the significant decrease in the GPS value (7.57° at the follow-up vs. 8.34° of baseline) indicates that after the training the kinematics of the gait pattern appeared closer to the physiological condition. As previously mentioned, there are indeed few studies that have investigated the effects of rehabilitative treatments on gait kinematics in individuals with PD (52–54) and none of them employ RAS as a tool to support gait training; thus, it is difficult to find data for comparisons. Moreover, the only two existing attempts to characterize the effects of RAS on kinematic patterns (30, 31) involved diseases different from PD (i.e., stroke and cerebral palsy). However, it is noteworthy that in both cases a significant reduction in the overall index of gait quality and, thus, a general improvement of the gait pattern was found, similar to what was observed in the present study.

**Figure 3:**
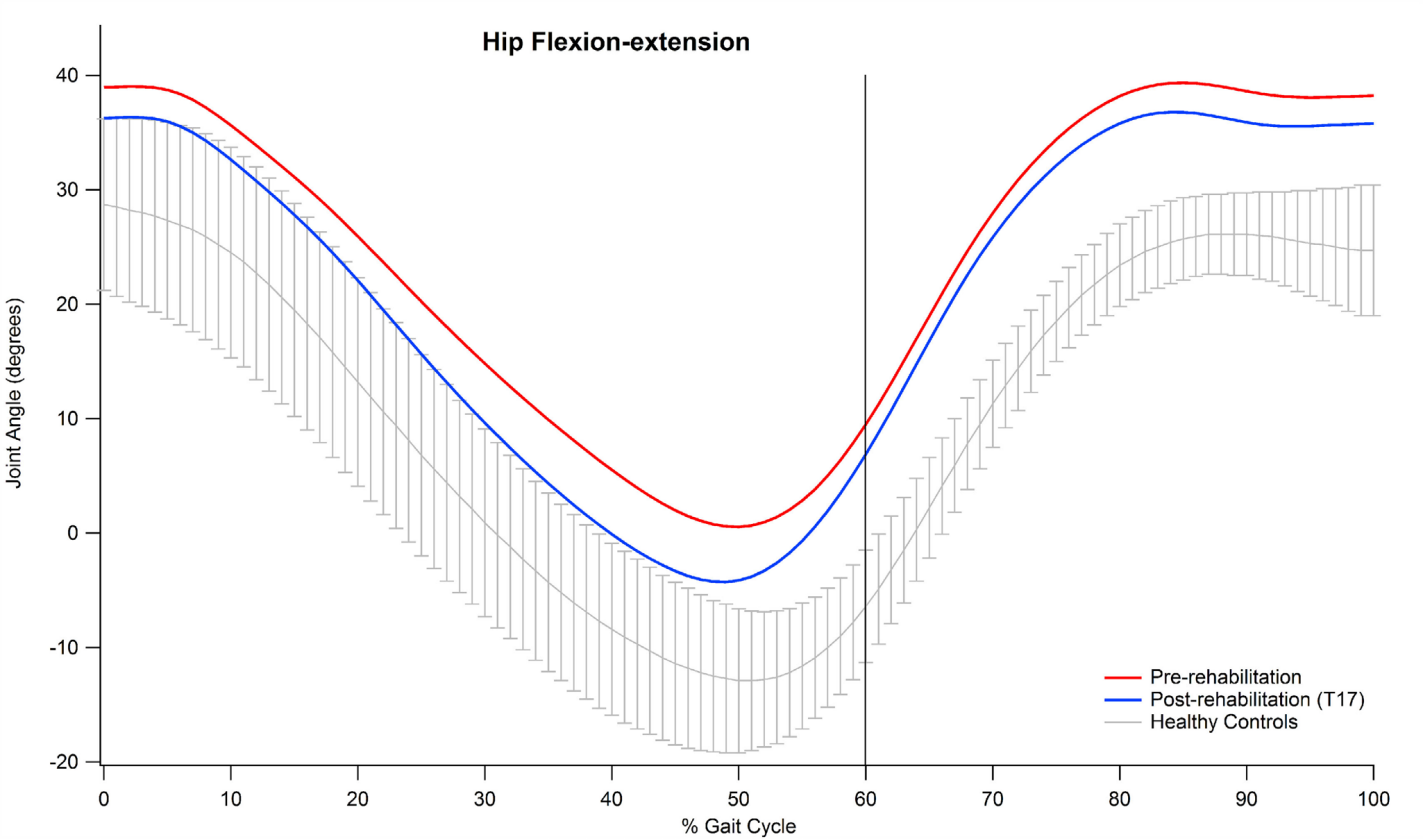
**Mean value of hip flexion–extension angle of participants pre and post-rehabilitation (T17) during gait**.

Examining the data of the present study in detail, it is interesting to observe that the major contribution to the improvement in the kinematic pattern of gait was essentially originated by a marked reduction in the hip flexion–extension GVS value and, to a lesser extent, in the knee flexion extension, as shown in Figure 2. In particular, the comparison between the hip flexion–extension angle during the gait cycle at the baseline and at the T17 follow-up (Figure 3) shows that the regularization of this movement is associated with a generalized decrease in flexion at heel contact and at the end of the swing phase, and with a correspondent increased extension at terminal stance.

Abnormal hip joint movements are quite common in neurological disorders as a compensation strategy for the lack of movement of the ankle joint (12), thus, it is likely that the positive effect of training integrated with RAS on the whole lower limb kinematic chain acts to recover a more physiological synergy between hip, knee, and ankle joint action. Moreover, our participants’ GVS score, associated with hip flexion–extension at baseline, was the largest in comparison with normality and, thus, it is likely that such a movement underwent more beneficial effects with respect to other joints which at the baseline resulted less impaired.

The large improvements originated in hip kinematics by the rehabilitative treatment probably represent one of the main factors responsible for the changes observed in the spatio-temporal parameters, particularly as regards step length and gait speed. In fact, previous studies observed that increases in hip ROM consequent to physical training are associated with increased step length (55) and that reduced peak hip extension is associated with a range of gait alterations, including reduced step length in both healthy subjects (45) and individuals affected by neurological diseases (56).

It was also surprising to observe that the GVS of ankle dorsi–plantarflexion was almost normal at the baseline, slightly (but not significantly) increased after rehabilitation, but significantly worse at the follow-up assessment, thus indicating a relevant deviation from normality. A possible explanation of this phenomenon can be found in the way that gait training is administered by physical therapists; to reduce the impact of existing (or future) shuffling gait and its negative consequences (i.e., slips, trips, and falls), the patient is stimulated to accentuate dorsiflexion at heel contact and the plantarflexion at toe off phase, thus making the movement a bit more “unnatural” as a preventive measure.

Other signs of improved gait kinematics come from the analyses of the dynamic ROM, which show that hip and knee ROM in the sagittal plane significantly increase after the training, while at ankle level no relevant changes were observed. These results are partly consistent with those of Kim et al. (30) who detected an increase in hip ROM of 6.4° after 9 sessions of gait training assisted by RAS (in our case 4.4° at the follow-up) and no significant changes as regards the ankle (similar to what was found in the present study). By contrast, after rehabilitation we found increases of ROM of the knee joint similar to those found by Kim et al. (2.1° vs. 3.2° in our study); however, they failed to achieve statistical significance, probably due to the limited size of their sample, which was composed of only 13 participants.

From a broader perspective, the present study further supports the efficacy of rehabilitation accompanied by RAS as a strategy to improve the gait parameters of Parkinson patients, thus confirming what was previously found in the literature [see Ref. (9, 12, 13)]. The major innovation of our study is that for the first time we report the effects of RAS not only on spatio-temporal parameters but also on gait kinematic variables. The original data reported herein are particularly important in gaining a better understanding of how the mechanics of gait are affected by auditory cues in this particular category of patients. Like every empirical work, the present study certainly has some limitations. The most important of these is the absence of a control group. Owing to the limited number of patients available in the hospital and to lack of space, we were able to test only one group of patients before and after the treatment. This prevents us from generalizing the results of the study and limits the possibility to assess whether the proposed treatment, which includes the gait training integrated by RAS, is superior in comparison with other kinds of rehabilitative approaches. Thus, even though we reported original information on patients’ gait kinematics, there is a need for future studies specifically focused on investigating the effects of RAS on gait kinematics in a randomized controlled trial.

Finally, it is noteworthy that although optoelectronic systems represent the most sophisticated option available for human movement analysis, they are expensive, require a dedicated laboratory (i.e., the whole equipment is not easily portable), and data acquisition and processing is time-consuming and can be performed only by specialized personnel. Future studies should consider other emerging techniques, such as wearable inertial sensors, which have been already successfully employed to characterize spatio-temporal parameters of gait in individuals with PD in clinical settings (57) but that can also be used to obtain data regarding the whole kinematic pattern (58).

## Conclusion

The overall analysis of gait patterns in individuals with PD before and after rehabilitation integrated with RAS – carried out taking into account not only the spatio-temporal parameters of gait but also the kinematic trends of lower limb joints – supplied new evidence about the effectiveness of such an approach. In particular, this technique appears capable of restoring several gait aspects to acceptable levels, thus making the ambulation function very similar to that of healthy individuals of the same age. In fact, it not only regularizes the cadence but also acts to increase speed and step length and creates a more physiological subdivision between stance and swing phase. As a plus, it is now recognized that this tool can influence gait kinematics, as the overall quality of gait pattern results significantly increased. However, from this point of view, some aspects still remain unclear. In fact, the positive kinematic effects of the training integrated with RAS appears basically restricted to the hip joint and are not always immediately visible after the end of the supervised treatment, but they rather tend to become evident after a longer period during which participants performed home-based gait training on a daily basis. The program also demonstrated limited effectiveness on knee joint functionality, which improved only in terms of dynamic ROM and, as regards the ankle, a slight (though significant) worsening of functionality was detected. As it appears quite consolidated that RAS has a positive effect on spatio-temporal aspects of gait, research should now focus on investigating kinematics in greater detail (but also kinetics and EMG variables that were also analyzed in a few studies) to better understand the mechanisms underlying gait disturbances in people with PD and, thus, establish new or improved rehabilitative treatments.

## Author Contributions

MP, MM, MG, CC, RP, FS, and TA designed the study; CC and RP performed the physical capacity assessment and prepared the rehabilitation protocol; GC performed the neurological evaluations; MP and FC collected and processed the data; MM and MG performed the statistical analyses; MP and MM wrote the manuscript; and TA, FS, GC, CC, and RP revised the manuscript.

## Conflict of Interest Statement

The authors declare that the research was conducted in the absence of any commercial or financial relationships that could be construed as a potential conflict of interest.

## Appendix

### A Rehabilitation Protocol

**Table:**

Targets	Exercises
Prevention of inactivity and fear of falling Prevention of falls Improving physical activity levels Recognizing the onset of fluctuations and adopting suitable movement strategies Learning simple motor exercises of increasing difficulty to be self-administered at home	Segmental exercises of active or assisted mobilization (flexion–extension, prono-supination) to increase strength, mobility, and coordination of the four limbs Stretching of anterior and posterior muscular kinetic chains Improvement of static balance: standing (uni- and bipedal), sitting, quadrupedal posture Improvement of dynamic balance: ambulation on paths of increasing levels of difficulty (e.g., turns, obstacles, etc.) Postural changes: from sitting/quadrupedal to standing, from supine/prone to lateral Occupational therapy exercises Gait training with RAS (for about 50% of the duration of each session)
